# Genome-wide association study of resistance to stripe rust (*Puccinia striiformis* f. sp*. tritici*) in Sichuan wheat

**DOI:** 10.1186/s12870-019-1764-4

**Published:** 2019-04-16

**Authors:** Xueling Ye, Jian Li, Yukun Cheng, Fangjie Yao, Li Long, Can Yu, Yuqi Wang, Yu Wu, Jing Li, Jirui Wang, Qiantao Jiang, Wei Li, Jian Ma, Yuming Wei, Youliang Zheng, Guoyue Chen

**Affiliations:** 1 Triticeae Research Institute, Sichuan Agricultural University, Wenjiang, Chengdu, Sichuan 611130 People’s Republic of China; 2 College of Agronomy, Sichuan Agricultural University, Wenjiang, Chengdu, Sichuan 611130 People’s Republic of China

**Keywords:** 55 K SNP, Genome-wide association study, Stripe rust, Resistance, Sichuan wheat accessions

## Abstract

**Background:**

Stripe rust (also called yellow rust) is a common and serious fungal disease of wheat (*Triticum aestivum* L.) caused by *Puccinia striiformis* f. sp. *tritici*. The narrow genetic basis of modern wheat cultivars and rapid evolution of the rust pathogen have been responsible for periodic and devastating epidemics of wheat rust diseases. In this study, we conducted a genome-wide association study with 44,059 single nucleotide polymorphism markers to identify loci associated with resistance to stripe rust in 244 Sichuan wheat accessions, including 79 landraces and 165 cultivars, in six environments.

**Results:**

In all the field assessments, 24 accessions displayed stable high resistance to stripe rust. Significant correlations among environments were observed for both infection (IT) and disease severity (DS), and high heritability levels were found for both IT and DS. Using mixed linear models, 12 quantitative trait loci (QTLs) significantly associated with IT and/or DS were identified. Two QTLs were mapped on chromosomes 5AS and 5AL and were distant from previously identified stripe rust resistance genes or QTL regions, indicating that they may be novel resistance loci.

**Conclusions:**

Our results revealed that resistance alleles to stripe rust were accumulated in Sichuan wheat germplasm, implying direct or indirect selection for improved stripe rust resistance in elite wheat breeding programs. The identified stable QTLs or favorable alleles could be important chromosome regions in Sichuan wheat that controlled the resistance to stripe rust. These markers can be used molecular marker-assisted breeding of Sichuan wheat cultivars, and will be useful in the ongoing effort to develop new wheat cultivars with strong resistance to stripe rust.

**Electronic supplementary material:**

The online version of this article (10.1186/s12870-019-1764-4) contains supplementary material, which is available to authorized users.

## Background

Wheat is an important food crop worldwide. However, wheat stripe rust caused by *Puccinia striiformis* f. sp. *tritici* (*Pst*) seriously threatens wheat production. Stripe rust is an airborne fungal disease, and *Pst* spores can be spread rapidly over long distances by the wind. Many wheat cultivars have become susceptible because newly developed races in the *Pst* population are virulent against the resistance genes in wheat cultivars. Stripe rust is a devastating disease in the cool, temperate wheat growing regions of the world, such as Asia, Europe, North America, South America, the Middle East and Africa [1]. China has the largest region in the world where stripe rust is endemic. The major endemic areas of stripe rust are the winter-wheat growing regions in the Northwest, Southwest and North of China, and the spring-wheat growing regions in Northwest China [2]. Sichuan Province in Southwest China is a hotspot for *Pst* to overwinter and oversummer. The *Pst* races CYR32 and CYR33 were detected in 1994 and 1997, and developed to predominant epidemic virulence levels in 2000 and 2007, respectively. This resulted in the loss of resistance to stripe rust in many cultivars, including Kangyin 655, Suwon 11 and Fan 6, as well as their derived cultivars [3]. Some cultivars retained resistance to CYR32 and CYR33 (e.g., cultivars with the gene *Yr24/26*, including Guinong 22 and its derived cultivars) and were planted as the main varieties in major regions of Sichuan Province. However, a new virulent pathogen, CYR34, appeared in 2009. In 2015, it became the main source of virulence against the *Yr24/26*-containing cultivars [4]. The variability of a pathogen is closely related to the evolution of host varieties. Breeding wheat cultivars for resistance to stripe rust is the most efficient, safe and environmentally sound strategy to control this disease.

Stripe rust resistance genes can be classified as race-specific and non-race-specific based on their effectiveness against different *Pst* races. A race-specific resistance gene confers resistance to a single race or many races, but is ineffective against other races. This resistance occurs in all growth stages, from seedling to adult stage. Therefore, it can be considered as seedling or all-stage resistance (ASR). Wheat cultivars with race-specific resistance genes have high-level resistance but may become susceptible when virulent races appear. In contrast, a non-race-specific resistance gene confers resistance to all races, Genes conferring adult-plant resistance (APR) are usually non-race-specific. High-temperature adult-plant resistance is a type of APR that is more effective at higher temperatures [5]. Because it is non-race specific, APR is usually durable, but often incomplete, and the resistance level is lower than that of ASR. Combining APR and ASR genes is the optimal method to develop cultivars with adequate durable resistance to stripe rust [6–8].

The use of a limited number of genetic stocks has decreased the genetic variation level in wheat breeding. Reduced diversity has become a bottleneck for stripe rust resistance improvement. To broaden the genetic basis of wheat cultivars, many resistance genes have been identified in wheat landraces and cultivars [9–23]. Wheat landraces have been shaped by traditional cultures and local cropping systems during long-term agricultural development. They adapt to different environments and collectively have a high diversity level and stable heritability [24]. Compared with cultivars, landraces have a greater diversity of genes that respond to abiotic and biotic stresses. Therefore, wheat landraces are valuable resources for stripe rust resistance breeding. The objectives of the present study were to (1) evaluate 244 Sichuan wheat accessions for resistance against *Pst* at the adult-plant stage in multiple years and field locations in Sichuan Province; (2) assess the genetic diversity, population structure and linkage disequilibrium (LD) pattern of the Sichuan wheat collection using the 55 K single nucleotide polymorphism (SNP) microarray; and (3) identify new stripe rust resistance loci using the genome-wide association study (GWAS) method.

## Results

### Phenotypic variation and diversity of stripe rust resistance

**Fig. 1:**
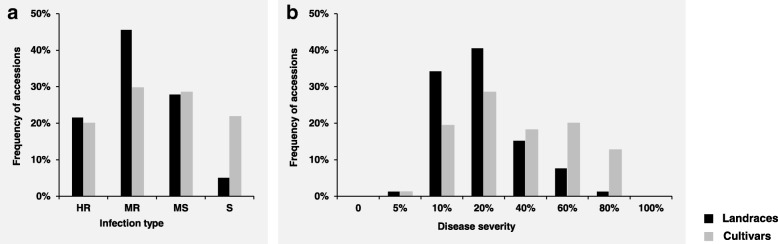
The distribution of infection type (IT) and disease severity (DS) for stripe rust based on BLU*P* values. **a** exhibited the IT distribution. **b** exhibited the DS distribution. IT, infection type; DS, disease severity; HR, highly resistance; MR, moderately resistance; MS, moderately susceptible; S, susceptible; The data are supplied in Additional file 2

The results of a correlation analysis revealed significant correlations among the six environments when IT and DS were separately analyzed at the adult-plant stage (Table 1). We identified 52 accessions that showed stable resistance to stripe rust in the six test environments. Among them, 24 accessions showed stable high-level resistance to stripe rust, including 12 landraces and 12 cultivars (Additional file 1). To reduce the environmental impacts on the stripe rust response, the best linear unbiased prediction (BLUP) values were calculated using a linear model with IT or DS in the six environments (Additional file 1). Based on the BLUP values, the IT distribution revealed that 53 landraces (67.09% of 79 landraces) exhibited stripe rust resistance; among them, 17 (21.52% of 79 landraces) landraces were highly resistant. For cultivars, 82 (49.70% of 165 cultivars) accessions showed resistance to stripe rust, and 33 (20.00% of 165 cultivars) cultivars were highly resistant. However, 26 (32.91% of 79 landraces) landraces and 83 (50.30% of 165 landraces) cultivars were susceptible to stripe rust (Fig. 1a; Additional file 2). Similarly, the DS distribution revealed that there were more resistant landraces than resistant cultivars (Fig. 1b; Additional file 2). The IT and DS data are summarized in Table 2. The mean IT and DS values of the landraces were lower than those of the cultivars in each test environment. When the environmental impact was removed from the BLUP values, the landraces still had higher resistance levels than did the cultivars. The mean IT values in landraces and cultivars were 2.16 and 2.52, respectively, and the mean DS values of landraces and cultivars were 23.23 and 37.15, respectively. Both IT and DS exhibited high heritability levels (0.91 and 0.90, respectively).

**Table 1:** The Pearson correlation analyses among six environments for IT (above diagonal) and DS (below diagonal) separately

Environments	CZ15	CZ16	MY16	CZ17	MY17	WJ17
CZ15	1	0.627**	0.600**	0.641**	0.597**	0.537**
CZ16	0.621**	1	0.705**	0.706**	0.627**	0.656**
MY16	0.644**	0.685**	1	0.672**	0.742**	0.700**
CZ17	0.585**	0.633**	0.693**	1	0.652**	0.730**
MY17	0.506**	0.512**	0.746**	0.658**	1	0.624**
WJ17	0.596**	0.662**	0.719**	0.709**	0.649**	1

*IT* infection type, *DS* disease severity, *CZ15* Chongzhou 2015, *CZ16* Chongzhou 2016, *MY16* Mianyang 2016, *CZ17* Chongzhou 2017, *MY17* Mianyang 2017, *WJ17* Wenjiang 2017

**, Significant at *p* < 0.01

**Table 2:** Summary of the stripe rust response between landraces and cultivars in six environments

Traits	Trials	Range		Mean		STDEV^a^		CV^b^		Heritability
		Landrace	Cultivar	Landrace	Cultivar	Landrace	Cultivar	Landrace	Cultivar	
IT^c^	CZ15	1-4	1-4	2.29	2.56	0.96	1.14	0.42	0.44	0.91
	CZ16	1–4	1–4	2.37	2.55	1.11	1.23	0.47	0.48	
	MY16	1–4	1–4	2.03	2.38	0.99	1.20	0.49	0.51	
	CZ17	1–4	1–4	2.00	2.32	0.82	1.18	0.41	0.51	
	MY17	1–4	1–4	1.56	2.29	0.97	1.21	0.62	0.53	
	WJ17	1–4	1–4	1.95	2.70	1.17	1.32	0.60	0.49	
	BLUP value^e^	1–4	1–4	2.16	2.52	0.82	1.05	0.38	0.41	
DS^d^	CZ15	0-100	0-100	30.06	47.61	25.16	35.20	0.84	0.73	0.90
	CZ16	0–80	0–100	23.67	43.19	20.17	31.34	0.85	0.73	
	MY16	0–100	0–100	27.03	38.69	26.99	36.91	1.00	0.95	
	CZ17	0–100	0–100	14.37	26.48	16.51	29.96	1.15	1.13	
	MY17	0–80	0–100	13.16	27.64	23.30	30.68	1.77	1.11	
	WJ17	0–100	0–100	10.82	33.06	20.36	35.33	1.88	1.07	
	BLUP value^e^	5–80	5–80	23.23	37.15	16.05	24.13	0.69	0.65	

^a^standard deviation

^b^coefficient of variation

^c^infection type

^d^disease severity

^e^the best linear unbiased prediction value

*CZ15* Chongzhou 2015, *CZ16* Chongzhou 2016, *MY16* Mianyang 2016, *CZ17* Chongzhou 2017, *MY17* Mianyang 2017, *WJ17* Wenjiang 2017

The 165 cultivars were collected from our institute and several other universities in Sichuan Province, and they were classified into four periods on the basis when they were bred. Periods 1, 2, 3, and 4 were from 1997 to 2001, 2002 to 2006, 2007 to 2011 and 2012 to 2016, respectively. There were 16, 55, 43, and 51 cultivars bred in periods 1, 2, 3, and 4, respectively (Additional file 1). The fewest cultivars were bred in period 1. Based on the analyses of BLUP values for IT, the cultivars bred in periods 2 (IT = 2.35) and 3 (IT = 2.35) displayed the highest resistance against stripe rust, while the cultivars bred in period 1 showed the lowest resistance (IT = 3.31). The cultivars bred in period 4 (IT = 2.61) showed moderate resistance. The Shannon–Weaver diversity index (*H′*) values were 0.80, 0.96, 0.98, and 0.98 for breeding periods 1, 2, 3, and 4, respectively (Table 3).

**Table 3:** The infection type (IT) diversity and genetic diversity analyses of cultivars in four periods

Statistics	Period 1	Period 2	Period 3	Period 4
Min	1	1	1	1
Max	4	4	4	4
Mean	3.31	2.35	2.35	2.61
*H′*	0.80	0.96	0.98	0.98
Gene Diversity	0.30	0.34	0.34	0.31
PIC	0.24	0.27	0.27	0.25

The periods 1–4 were from 1997 to 2001, 2002 to 2006, 2007 to 2011 and 2012 to 2016, respectively

*H‘*, Shannon-Weaver diversity index

PIC, polymorphism information content index

### Genetic diversity analysis

A total of 44,059 SNP markers were obtained for the 244 accessions based on the criteria of missing values ≤10% and minor allele frequency (MAF) ≥ 5%. Among them, 16,026, 16,770 and 11,263 SNP markers were mapped on sub-genomes A, B and D, respectively (Table 4). The map lengths of sub-genomes A, B and D were 4930.66, 5177.04 and 3947.75 million base pairs (Mb), respectively, and the average marker densities for the three sub-genomes were 3.3, 3.3 and 2.9 markers per Mb, respectively. Chromosome 6A had the highest marker density at 4.0 markers per Mb, while chromosome 4D had the least markers (944) and had the lowest marker density at 1.9 markers per Mb. Among the three sub-genomes, sub-genome B showed the lowest major allele frequency (0.695), highest gene diversity (0.396), and highest polymorphism information content index (PIC) (0.312) values. Sub-genome D exhibited the highest major allele frequency (0.719), lowest gene diversity (0.369), and lowest PIC (0.293) values. Among the chromosomes, chromosome 4A showed the highest major allele frequency (0.778), lowest gene diversity (0.316), and lowest PIC (0.259) values, while chromosome 5B exhibited the lowest major allele frequency (0.665), greatest gene diversity (0.422), and highest PIC (0.329) values.

**Table 4:** Summary of genetic diversity of 244 wheat accessions on sub-genomes and chromosomes

Chr.^a^	No. of markers	Map length (Mb)	No. of markers per Mb	Major Allele Frequency	Gene Diversity	PIC^b^
1A	2183	593.28	3.7	0.706	0.385	0.305
2A	2547	780.76	3.3	0.712	0.391	0.311
3A	2003	750.63	2.7	0.706	0.383	0.303
4A	2174	743.32	2.9	0.778	0.316	0.259
5A	2260	709.22	3.2	0.684	0.404	0.317
6A	2456	617.99	4.0	0.745	0.343	0.276
7A	2403	735.46	3.3	0.687	0.401	0.316
Sub-genome A	16,026	4930.66	3.3	0.717	0.375	0.298
1B	2418	688.70	3.5	0.670	0.419	0.327
2B	2376	801.23	3.0	0.714	0.379	0.302
3B	2411	829.73	2.9	0.703	0.384	0.304
4B	2401	673.47	3.6	0.725	0.371	0.296
5B	2429	712.40	3.4	0.665	0.422	0.329
6B	2436	720.96	3.4	0.696	0.395	0.312
7B	2299	750.55	3.1	0.688	0.401	0.315
Sub-genome B	16,770	5177.04	3.3	0.695	0.396	0.312
1D	1815	495.23	3.7	0.685	0.406	0.318
2D	1926	651.04	3.0	0.743	0.354	0.284
3D	1545	615.49	2.5	0.713	0.377	0.300
4D	944	509.27	1.9	0.757	0.329	0.266
5D	1539	564.75	2.7	0.700	0.386	0.304
6D	1528	473.52	3.2	0.741	0.350	0.281
7D	1966	638.46	3.1	0.696	0.382	0.300
Sub-genome D	11,263	3947.75	2.9	0.719	0.369	0.293

^a^chromosome

^b^polymorphism information content index

**Fig. 2:**
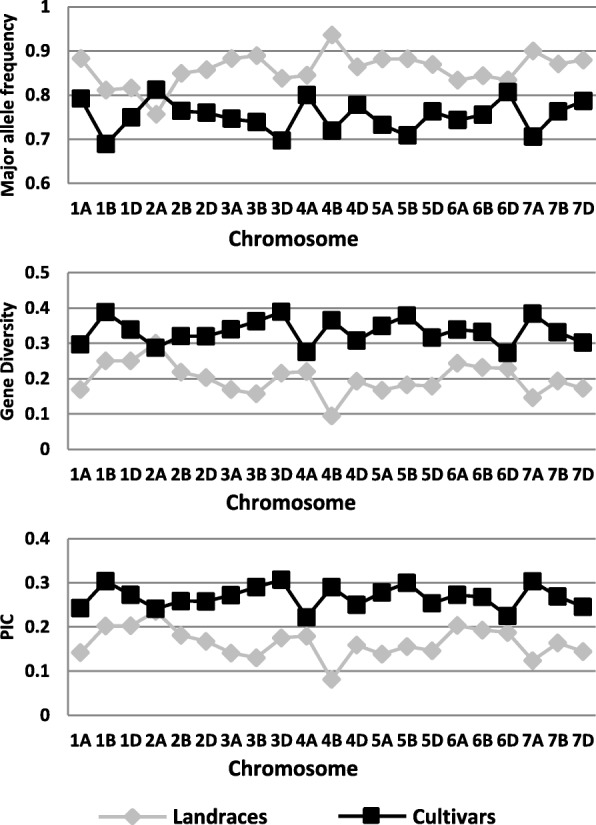
The genetic diversity between landraces and cultivars using SNP markers. SNP, single nucleotide polymorphism; PIC, polymorphism information content index; The data are supplied in Additional file 3

There were significant differences in genetic diversity between landraces and cultivars (Fig. 2; Additional file 3). The major allele frequencies were significantly higher in landraces than in cultivars among the three sub-genomes and 21 chromosomes, except for chromosome 2A. The gene diversity and PIC values of cultivars were significantly higher than those of landraces among the three sub-genomes and 21 chromosomes, except for the gene diversity of chromosome 2A.

There were differences in genetic diversity among the four breeding periods. The cultivars bred in periods 2 and 3 exhibited the highest gene diversity (0.34) and PIC (0.27) values. The cultivars bred in period 1 had the lowest gene diversity (0.30) and the lowest PIC (0.24) values. The cultivars bred in period 4 had medium diversity (0.31) and PIC (0.25) values (Table 3).

### Population structure, kinship and LD analyses

**Fig. 3:**
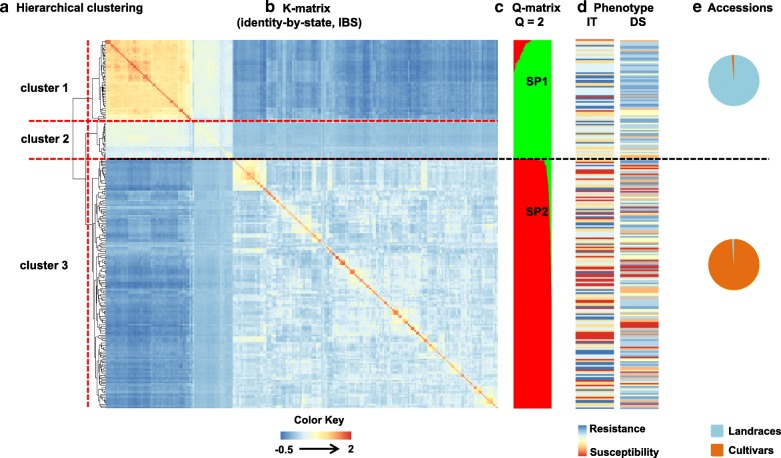
Population structure of 244 Sichuan wheat accessions. **a** Hierarchical clustering based on Ward method with K-matrix indicated the 244 accessions could be divided into three clusters (cluster 1, cluster 2 and cluster 3). **b** Heat map of the IBS (identity by state) relative K-matrix. **c** The population structure of 244 accessions with Bayesian clustering analysis. Two colors stand for 2 different compositions. Different color area of each line represented different proportion of composition. The Sub-population 1 (SP1) mainly showed as green color. The Sub-population 2 (SP2) mainly showed as red color. **d** Heat map of IT and DS (IT, infection type; DS, disease severity) with BLUP (best linear unbiased prediction) values across six environments in the field. Blue to yellow to red lines show resistance to intermediate to susceptibility to stripe rust of the corresponding accessions. **e** Percentage memberships of accessions from two subpopulations based on Q-matrix. The landraces showed as blue color and cultivars showed as red color. The red dashed divided the K-matrix into three parts according the hierarchical clustering and the black dashed divided the items into two parts according the Bayesian clustering

The population structure (Q-matrix) was calculated using the 44,059 SNP markers for 244 accessions based on the delta K (ΔK) method of Bayesian clustering. The accessions were classified into two sub-populations, 1 (SP1) and 2 (SP2) (Fig. 3). In total, 78 and 166 accessions were included in SP1 and SP2, respectively. All the landraces were classified in SP1, except AS661599 and AS1679, which were included in SP2. All the cultivars were classified in SP2, except for one cultivar Xifu14, which was included in SP1. According to the mean BLUP-based IT and DS values, the landraces in SP1 had higher resistance levels (IT = 2.14, DS = 22.50) than the cultivars in SP2 (IT = 2.53, DS = 37.41) (Fig. 3; Additional file 4). In addition, the cultivars in SP2 showed higher genetic diversity (PIC = 0.27, gene diversity = 0.33) and higher phenotypic diversity indexes (*H′* = 0.99 for IT and *H′* = 0.75 for DS). The landraces in SP1 showed lower genetic diversity (PIC = 0.15, gene diversity = 0.19) and lower phenotypic diversity indexes (*H′* = 0.84 for IT and *H′* = 0.58 for DS) (Additional file 4).

To understand the genetic relationships among the 244 accessions, an identity-by-state relative kinship matrix (K-matrix) was estimated. The heat map of the K-matrix is shown in Fig. 3. Unlike the Bayesian clustering analysis with the Q-matrix, the hierarchical clustering based on the Ward method with the K-matrix divided the 244 accessions being divided into three clusters. There were 54, 25, and 165 accessions in clusters 1, 2, and 3, respectively. As shown in Fig. 3, the landraces classified as SP1 by Bayesian clustering were further classified as clusters 1 and 2 by hierarchical clustering. Relatively, cluster 2 comprised cultivars, except for cultivars Yumai 1 and Xifu 14, which were assigned to cluster 1.

**Fig. 4:**
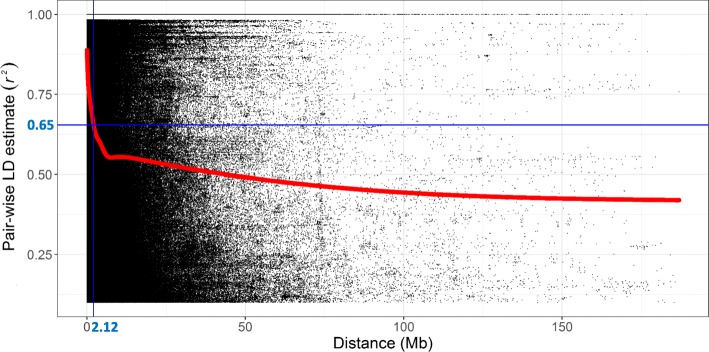
Genome-wide average linkage disequilibrium (LD) decay over physical distances based on 44,059 SNP markers. The red curve represents the model fits to LD decay. The blue line represents the half LD decay distance was 2.12 Mb when the LD declined to 50% (*r*^*2*^ = 0.65) of its initial value. Mb, million base pairs

The pairwise measure of LD was estimated based on the allele frequency correlations (*r*^*2*^) between significant pairs of intra-chromosomal SNP markers with physical distances (Fig. 4). The half-decay distance was 2.12 Mb when the LD declined to 50% (*r*^*2*^ = 0.65) of its initial value. We defined the significant associated loci on the same chromosome within the 2.12-Mb genomic region as being in the same QTL block.

### Genome-wide association analyses

**Fig. 5:**
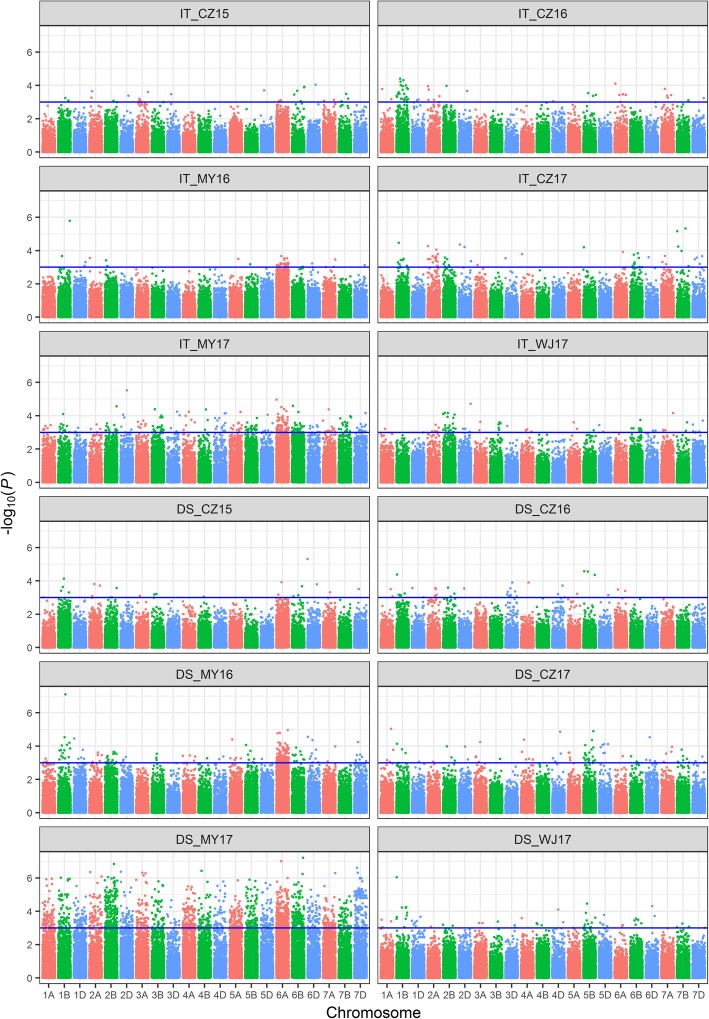
The *P* values of associated loci with infection type (IT) and disease severity (DS) displayed as Manhattan plots. The associated loci with IT and DS in six environments were displayed as Manhattan plots with *P* values across 21 wheat chromosomes. The significant associated locus was considered as –log_10_(*P*) > 3 which upper the blue lines. IT, infection type; DS, disease severity. CZ15 = Chongzhou 2015, CZ16 = Chongzhou 2016, MY16 = Mianyang 2016, CZ17 = Chongzhou 2017, MY17 = Mianyang 2017, WJ17 = Wenjiang 2017

Based on the mixed linear model analysis with Q + K as covariates using 44,059 SNP markers, GWASs were performed for the IT and DS of stripe rust against the *Pst* populations within each of the six environments at the adult-plant stage. There were 7 and 12 high-confidence loci associated with IT and DS at *P* < 0.001, and they were located on the long arms of chromosomes 1B, 3D, 5A, 5B, and 7B and the short arms of chromosomes 1A, 5A, 6A, 6B, and 7A (Table 5). The loci linked to *AX-111488534* were associated with both IT and DS, and the phenotypic variation explained (PVE) was up to 13.6 and 17%. Additionally, we detected 18 high-confidence loci associated with resistance to stripe rust. The high-confidence loci in the six test environments are displayed as Manhattan plots with *P* values across 21 wheat chromosomes in Fig. 5. Furthermore, 12 QTLs were identified according to the half-decay distance 2.12 Mb. Chromosomes 1BL, 5A and 5BL contained two QTLs each. Also, one QTL was identified on each of chromosomes 1AS, 3DL, 6AS, 6BS, 7AS, and 7BL. Two of the QTLs were potentially novel based on their unique chromosomal locations when referenced to the consensus [25] and physical [26] maps.

**Table 5:** The summary of high confidence associated loci

QTL name	SNP Marker	Chr.^a^	Position	Allele	Trait	-log_10_(*P* values)	Marker R^2^ (%)	Environments^b^
*Qyrsicau-1AS*	AX-110375459	1A	55,931,144	C/T	DS	3.2–4.0	7.4–8.4	MY16, CZ17, MY17
*Qyrsicau-1BL.1*	AX-108839316	1B	670,373,135	T/C	DS	3.1–4.2	6.4–9.1	MY16, MY17, WJ17
	AX-109440891	1B	670,379,326	T/A	DS	3.1–4.1	6.4–7.9	MY16, MY17, WJ17
	AX-109389405	1B	670,382,321	T/C	DS	3.4–4.0	7–9.5	MY16, MY17, WJ17
	AX-108930953	1B	670,418,012	C/T	DS	3.9–4.2	8.1–10	MY16, MY17, WJ17
	AX-108800083	1B	670,502,837	A/G	DS	3.2–4.3	5.3–7.2	MY16, MY17, WJ17
	AX-111488534	1B	670,550,099	T/C	IT & DS	4.1–5.8 & 3.1–7.1	6.9–13.6 & 7.6–17.0	CZ16, MY16, CZ17, MY17, WJ17
	AX-109335890	1B	670,593,327	A/C	IT	3.1–4.2	5.1–8.8	CZ16, CZ17, MY17
*Qyrsicau-1BL.2*	AX-111471952	1B	681,682,184	A/G	IT	3.0–4.4	6.7–9.7	CZ16, CZ17, MY17
*Qyrsicau-3DL*	AX-109329567	3D	595,680,421	A/G	IT	3.4–4.0	8–10.1	CZ15, CZ17, MY17
Qyrsicau-5AS*	AX-111623511	5A	101,053,846	A/G	IT	3.5–4.2	8.1–9.7	MY16, MY17, WJ17
Qyrsicau-5AL*	AX-109907430	5A	566,772,733	T/C	DS	3.0–4.4	6.1–10.4	CZ16, MY16, WJ17
*Qyrsicau-5BL.1*	AX-111051502	5B	554,577,408	C/T	DS	3.3–4.1	6.7–9.4	MY16, MY17, WJ17
*Qyrsicau-5BL.2*	AX-110123238	5B	589,800,894	G/C	DS	3.4–3.6	7.7–8.8	MY16, CZ17, MY17
*Qyrsicau-6AS*	AX-108943926	6A	24,724,553	A/G	DS	3.3–3.9	7.2–9.5	CZ15, MY16, MY17
*Qyrsicau-6BS*	AX-109937061	6B	28,634,217	C/T	DS	3.5–6.2	7.4–13.3	MY16, MY17, WJ17
*Qyrsicau-7AS*	AX-109348608	7A	81,528,245	G/A	IT	3.1–3.6	6.8–8.2	CZ15, CZ17, WJ17
*Qyrsicau-7BL*	AX-110518451	7B	711,405,854	G/A	IT	3.1–5.3	6.4–11.3	CZ16, CZ17, MY17

^a^chromosome

^b^the environments where the high confidence associated marker can be identified in

^*^ the potential novel QTL

CZ15 = Chongzhou 2015, CZ16 = Chongzhou 2016, MY16 = Mianyang 2016, CZ17 = Chongzhou 2017, MY17 = Mianyang 2017, WJ17 = Wenjiang 2017

### Favorable allele analyses

There were 18 SNP markers significantly associated with stripe rust in at least three environments. Of them, 12 favorable alleles were identified based on significant differences in IT and DS between the accessions with and without favorable alleles as detected by *t*-test. According to the total frequency calculation, the landraces contained more favorable alleles than did the cultivars (Table 6; Additional file 5). The correlation analyses revealed a significant negative correlation between favorable allele number and the reaction against stripe rust (Additional file 6). The materials with more favorable alleles, and they exhibited higher resistance to stripe rust. In contrast, those with fewer favorable alleles had weaker resistance (or higher susceptibility). These results support the use of a combination of several loci for wheat disease resistance breeding.

**Table 6:** Summary of favorable allele in 244 wheat accessions

SNP marker	Alleles	Chr.^a^	Position (bp)	Associated traits	Favorable alleles	*t*-test^b^	Frequency	
							Landrace	Cultivar
AX-110375459	C/T	1A	55,931,144	DS	TT	*	0.30	0.07
AX-108930953	C/T	1B	670,418,012	DS	CC	*	0.05	0.73
AX-111488534	T/C	1B	670,550,099	IT & DS	CC	* & ***	0.90	0.05
AX-109329567	A/G	3D	595,680,421	IT	GG	***	0.01	0.09
AX-111623511	A/G	5A	101,053,846	IT	AA	***	0.59	0.32
AX-109907430	T/C	5A	566,772,733	DS	CC	**	0.38	0.17
AX-111051502	C/T	5B	554,577,408	DS	TT	******	0.89	0.15
AX-110123238	G/C	5B	589,800,894	DS	CC	*	0.11	0.42
AX-108943926	A/G	6A	24,724,553	DS	GG	*	0.43	0.23
AX-109937061	C/T	6B	28,634,217	DS	TT	**	0.20	0.47
AX-109348608	G/A	7A	81,528,245	IT	GG	***	0.25	0.64
AX-110518451	G/A	7B	711,405,854	IT	GG	*****	0.06	0.33
Total frequency							4.19	3.67

IT, infection type; DS, disease severity

^a^, chromosome

^b^, significant difference in IT or/and DS between accessions with and without favorable alleles

*, significant at *P* < 0.5; **, significant at *P* < 0.01; ***, significant at *P* < 0.001; *****, significant at *P* < 0.00001; ******, significant at *P* < 0.000001

## Discussion

### Phenotypic and genetic diversity

To identify the stripe rust resistance genes in our materials, we used a uniform mixture of seven *Pst* races to infect 244 accessions in the field. The seven *Pst* races were the predominant races in China at present, of which CYR34 (V26) is the latest race virulent to *Yr24* (*= Yr26* and *YrCH42*). In total, 12 landraces (15.19% of 79 landraces) and 12 (7.27% of 165 cultivars) cultivars showed stable high resistance to stripe rust in all the test environments. A higher percentage of landraces than cultivars exhibited resistance to stripe rust. The BLUP values were calculated to reduce the environmental impact. The mean IT and DS values also revealed that the landraces had higher resistance than did the cultivars, independent of the environment and when using BLUP values (Table 2). In addition, the frequency of favorable alleles was higher in landraces than in cultivars (Table 6; Additional file 5). Thus, the landraces contained abundant resistance genes and were identified as the elite germplasms for stripe rust resistance breeding. The wheat landraces are the outcome of natural selection, and reportedly have rich elite genes and a good affinity for hybridization. Chinese Spring is a well-known landrace derived from China (Sichuan Province) that is used as the international reference (IWGSC, http://www.wheatgenome.org/). The APR and pleiotropic gene *Lr34/Yr18* has maintained stable resistance to multiple fungal pathogens for a long time. It was derived from Chinese landraces and is widely used in modern wheat cultivars [14, 16, 25, 27–29]. In addition, many stripe rust resistance genes have been identified in landraces, such as *Yr45* [17], *Yr52* [18], *Yr53* [30], *Yr59* [31], *Yr62* [32], *Yr64* [33], *Yr65* [33], *Yr72* [34] and *Yr79* [22] as well as many other genes and QTLs [25].

Although more landraces than cultivars were resistant to stripe rust, the cultivars from Sichuan Province showed good resistance. In total, 82 (49.70% of 165 cultivars) cultivars showed resistance against stripe rust and maintained resistance for several years, as a result of the use of resistance genes in breeding. In addition, genotype analyses revealed that the cultivars had higher genetic diversity and more phenotypic variation than did the landraces, as assessed by the 44,059 SNP markers (Fig. 2; Additional file 3). We classified the materials into four periods according to breeding years. The analyses of phenotype and genotype showed that the cultivars bred in period 1 had the lowest *H′* (0.80), resistance (IT = 3.31), gene diversity (0.30), and PIC (0.24) values. While those bred in periods 2–4 showed significantly higher resistance, *H′*, gene diversity, and PIC values (Table 3). The phenotypic diversity index also increased in more recent breeding years. The cultivars in period 1 were bred between 1997 and 2001, when were few superior stocks for breeding. Consequently, fewer accessions were bred in those years. The limited breeding stocks resulted in less diversity and a narrow genetic background, which caused a breeding bottleneck. We speculated about the reasons for the significant differences between period 1 and the other three periods. One possible explanation is the ‘Cooperative China/International Maize and Wheat Improvement Centre shuttle Breeding Program’, which was signed in 1987 [35]. Sichuan Province was one of three designated shuttle breeding areas, but it took about 10 years to exchange information and germplasms to prepare for breeding. Therefore, the breeding of new cultivars containing foreign germplasm in their pedigrees started in about 2000 in Sichuan Province [36]. This would explain the obvious differences in stripe rust resistance, phenotypic variation, and genetic diversity between period 1 and the other periods (periods 2–4) (Table 3). The introduction of new resources is an efficient strategy to improve genetic diversity and broaden the genetic basis. Thus, landraces, as a major resource for resistance to stripe rust, are elite germplasms for wheat breeding.

### Population structure and genetic relationships

Whether the population structure was established by a Bayesian model-based clustering or a Ward method-based genetic relationship with the K-matrix, the cultivars were mainly classified in the same cluster and the landraces were mainly classified in other clusters (Fig. 3). The significant differences in phenotypic variation and genetic diversity supported the classification based on the Q-matrix, which demonstrated the relatively distant genetic relationships between landraces and cultivars. The accessions in SP1 had higher resistance to stripe rust, lower phenotypic diversity and lower genetic diversity values than accessions in SP2 (Additional file 4). Compared with Bayesian clustering, the hierarchical clustering classified the cultivars into one cluster, cluster 3, but further divided the landraces into two clusters (clusters 1 and 2). The hierarchical clustering also indicated the differences in genotyping between landraces and cultivars (Fig. 3). These results indicated that the landraces in this study are the elite germplasms that can be used to broaden the genetic background and improve genetic diversity.

### Comparison of high-confidence loci with reported yellow rust resistance (*Yr*) genes and QTLs

In total, 18 high-confidence loci were detected. Based on the half-decay distance of 2.12 Mb, we classified the 18 high-confidence loci into 12 QTLs, which were located on 1AS, 1BL, 3DL, 5AS, 5AL, 5BL, 6AS, 6BS, 7AS and 7BL. The QTLs were named as follows: *Qyrsicau-1AS*, *Qyrsicau-1BL*, *Qyrsicau-3DL*, *Qyrsicau-5AS*, *Qyrsicau-5AL*, *Qyrsicau-5BL*, *Qyrsicau-6AS*, *Qyrsicau-6BS*, *Qyrsicau-7AS*, and *Qyrsicau-7BL* (Table 5).

*Qyrsicau-1AS* was located at 55.9 Mb in the distal region of 1AS, which was covered by *QYr.sgi-1A.1* [37]. *Qyrsicau-1BL.1* and *Qyrsicau-1BL.2* were located at 670 Mb and 681 Mb, respectively. The marker *AX-111488534* linked to *Qyrsicau-1BL.2* explained up to 17.0% PVE and was associated with both IT and DS. There were four reported *Yr* genes and more than 10 QTLs near this region. *Qyrsicau-1BL.1* was close to *Yr29* and overlapped with *Qyrsun-1BL* [38]. Additionally, *Qyrsicau-1BL.2* was very close to *QYr.tam-1B*, which was linked to *XwPt-668,027* [39] according to the physical map IWGSC RefSeq v1.0 [26].

In group 3 chromosomes, only one QTL (*Qyrsicau-3DL*) was located at 3DL. The associated marker *AX-109329567* was located at 595 Mb, and the PVE ranged from 8.0 to 10.1%. *Yr71* on 3DL was identified as an *APR* gene and was located 1.8 cM from marker *Xgwm114b*/*KASP_8306*/*KASP_17207*/*KASP_16434* [40], which was close to *Qyrsicau-3DL*.

**Fig. 6:**
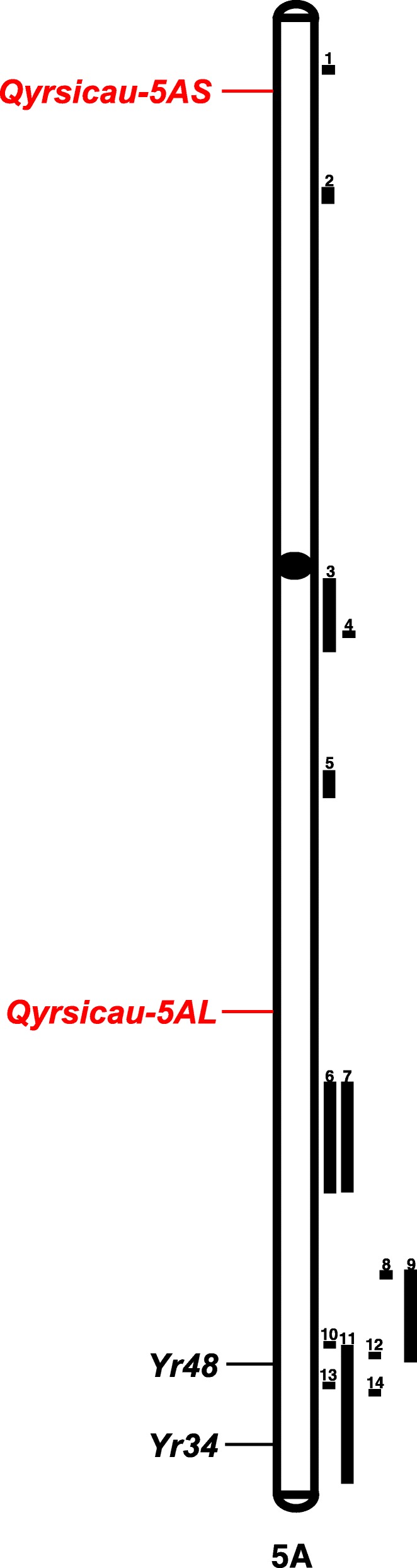
The position of the potentially novel QTLs on the chromosome in this study. The chromosome lengths were all standardized to the same relative length. QTLs marked as red color on the left side of chromosomes were the potentially new QTLs in this study. The reported genes and QTLs were marked as black color and mapped on the left and right side of the chromosomes separately which referred Wang and Chen (2017) [25]

There were four QTLs located in group 5 chromosomes, two on chromosome 5A and two on chromosome 5B. *Qyrsicau-5AS* was located at the distal region of 5AS (Fig. 6). There were two QTLs reported at 5AS. The QTL linked with *wsnp_Ex_c807_1586396-5AS* [41] was identified as an *APR* and was ~ 80 Mb from *Qyrsicau-5AS*. Another QTL, *QYr.cau-5AS* [42] was a major QTL that exhibited a high PVE, but *Qyrsicau-5AS* was a minor QTL with a PVE of 7.1 to 8.1%. Two previously reported QTLs at 5AS were different from *Qyrsicau-5AS* identified in our research, suggesting that *Qyrsicau-5AS* is a novel QTL. Another QTL on 5A, *Qyrsicau-5AL* (Fig. 6), was identified as a potential novel QTL located between *QYrtb.pau-5A* [43] and *QYR5-5A* [44] on the consensus map [25]. Another two QTLs were located at the middle region of 5BL. *Qyrsicau-5BL.1* had a PVE of 9.4%, and *Qyrsicau-5BL.2* had a PVE of 8.8%. *Qyrsicau-5BL.1* was mapped at 554 Mb on 5BL and was very close to the QTL [41] linked to marker *wsnp_Ex_c2582_4804223*. Another QTL on 5BL was *Qyrsicau-5BL.2*, which was covered by *QYr.tem-5B.2* [45].

Two QTLs were located in group 6 chromosomes. *Qyrsicau-6AS* was located at the end of 6AS and covered by *QYr.wgp-6AS* [46]. *Qyrsicau-6BS* was located at the distal end of 6BS and was very close to previously reported *QYr.ufs-6B* [37].

The group 7 chromosomes contained 2 QTLs. *Qyrsicau-7AS* was mapped at the 81-Mb position on chromosome 7AS and was covered by the known *QYr.inra-7A* [47] and *QYr.sun-7A* [48] based on the consensus map [25]. *Qyrsicau-7BL* was associated with *AX-110518451*, which was covered by *QYr.caas-7BL.2* [49], and the PVE ranged from 6.4 to 11.3%.

Finally, six QTLs were covered by reported genes or QTLs. Four QTLs were very close to reported genes or QTLs, and two QTLs were identified as potential novel QTLs. Because of the lack of markers in common with the consensus map, and because only the rough locations of some reported genes and QTLs are known, these QTLs need to be analyzed in more detail. Allelism tests and fine mapping are effective ways to identify real novel resistance genes in future studies.

### Analyses of putative candidate genes in two potential novel QTLs

Based on the Chinese Spring reference RefSeq v1.0 (IWGSC) and RefSeq Annotation v1.1 [26], 12 and 36 genes were identified within the *Qyrsicau-5AS* and *Qyrsicau-5AL* regions, respectively. By referencing the disease resistance-related signaling pathway, resistance-related protein family, and genes reported previously, nine candidate genes (Additional file 7) related to stripe rust resistance were identified, which were homologous to those in *Arabidopsis* (*Arabidopsis thaliana*), rice (*Oryza sativa* L. ssp. *japonica*) and maize (*Zea mays*).

One putative candidate gene, *TraesCS5A02G079700*, was detected in *Qyrsicau-5AS* and orthologous to *Os03g0144800* in rice (*O. sativa* subsp. *japonica*) and *MUR3* (xyloglucan galactosyltransferase) in *Arabidopsis*. Both these genes belong to the glycosyltransferase 47 family and are involved in the salicylic acid-mediated signaling pathway [50]. Salicylic acid is a plant hormone and that functions as a signaling molecule in systemic immunity in response to pathogen infection [51].

Eight presumptive candidate genes (*TraesCS5A02G364700*, *TraesCS5A02G365300*, *TraesCS5A02G365600*, *TraesCS5A02G365700*, *TraesCS5A02G365800*, *TraesCS5A02G367100*, *TraesCS5A02G367700* and *TraesCS5A02G367900*) were present in *Qyrsicau-5AL*. *TraesCS5A02G364700*, *TraesCS5A02G365300*, *TraesCS5A02G365600* and *TraesCS5A02G365700* were all homologous to the wall-associated receptor kinase *WAK* and to *At5g49770* in *Arabidopsis*. *WAK* belongs to the serine/threonine (Ser/Thr) protein kinase family, and it is involved in defense responses to fungal infections and to salicylic acid [52, 53]. The other gene, *At5g49770*, also belongs to the Ser/Thr protein kinase family. This gene is a leucine-rich repeat receptor-like protein kinase, which may be related to disease resistance [54–56]. The *TraesCS5A02G365800* orthologous genes, *KDEL*-tailed cysteine endopeptidase *CEP1*, cysteine proteinase *RD21A*, and senescence-specific cysteine protease *SAG12*, which all belong to the peptidase C1 family and are involved in defense responses to fungal infections [57–59]. Furthermore, cysteine protease plays an important role in immunity and is involved in elicitor-stimulated programmed cell death [60]. The symptoms in infected wheat include the appearance of hypersensitive flecks or necrosis, which are characteristics of programmed cell death. *TraesCS5A02G367100* is orthologous with the *Arabidopsis* genes *BAM1* (derived from barley any meristem 1), *BAM2*, *BAM3* receptor-like kinases, and *RLK5* (receptor-like protein kinase 5), the rice gene *FON1* (floral organ number 1), and the maize gene *TD1* (thick tassel dwarf 1). All of these genes belong to the Ser/Thr protein kinase family and contain a disease resistance-related domain (leucine-rich repeat receptor-like protein kinases). *TraesCS5A02G367700* is homologous to the rice gene *Os12g0486900*, and the *Arabidopsis* genes *At4g00960*, *LRK10L-2.4*, and *PR5K* (pathogenesis-related protein 5-like receptor kinase). They also belong to the Ser/Thr protein kinase family. In particular, the gene *LRK10L-2.4* is a leaf rust resistance locus and a receptor-like gene in *Arabidopsis* [61]. *TraesCS5A02G367900* is aligned with rice gene *Os03g0670100* and the *Arabidopsis* gene *TGD3* (trigalactosyldiacylglycerol 3), which contain ATP-binding cassette (ABC) transporters. We considered this gene as a candidate because *Yr18/Lr34* conferring APR also contains an ABC transporter [62].

The candidate gene analyses were limited by the use of the Chinese Spring reference genome because dispensable genes or variations exist among individual genotypes within and between species. However, the collinear alignment analyses have provided us with important clues to identify candidate genes in our accessions. We will study these putative candidate genes by reverse genetics in future studies.

## Conclusions

To use multiple resistance loci to breed for stable and durable resistance to stripe rust, more and new resistance loci need to be discovered. Wheat landraces are elite germplasms for the exploration of new resistance genes. Here, we identified 24 accessions harboring resistance genes that showed stable high-level resistance in six test environments. The GWAS results revealed 12 QTLs associated with 18 SNP markers. Among them, there were 12 favorable alleles for stripe rust resistance and two potentially novel loci. Finally, we predicted nine candidate genes related to stripe rust resistance. Our study provides SNP markers associated with resistance loci that will be useful for marker-assisted selection in wheat breeding.

## Methods

### Plant materials

A collection of 244 Sichuan wheat accessions was assembled mainly from germplasm bank accessions collected and stored at the Triticeae Research Institute, Sichuan Agricultural University (germplasm numbers abbreviated AS) and the Chinese Crop Germplasm Resources Bank (germplasm numbers abbreviated ZM), China. We analyzed a total of 79 Sichuan wheat landraces and 165 commercial varieties (Additional file 1), which had been derived from different breeding units (Sichuan Academy of Agricultural Sciences, Mianyang Academy of Agricultural Sciences, Neijiang Academy of Agricultural Sciences, Chengdu Institute of Biology, Chinese Academy of Sciences, Sichuan Agricultural University and Southwest University of Science and Technology) in Sichuan Province since 1997. The 165 cultivars were bred between 1997 and 2016. We classified them into four 5-years breeding periods according to the time they were bred. Periods 1–4 were from 1997 to 2001, 2002 to 2006, 2007 to 2011, and 2012 to 2016, respectively.

### Phenotyping for stripe rust resistance in field environments

The 244 accessions were tested for field stripe rust responses at the adult stage after artificial inoculation in the following six environments in Sichuan: Chongzhou (30°33′N 103°39′E) in 2015, 2016 and 2017; Mianyang (31°23′N 104°49′E) in 2016 and 2017, and Wenjiang (30°43′N 103°52′E) in 2017, referred to as CZ15, CZ16, MY16, CZ17, MY17 and WJ17, respectively. In all test environments, 20 seeds of each accession were planted in rows 2-m in length spaced 30-cm apart, with individual plants spaced 10-cm apart. Susceptible wheat varieties SY95–71 and Taichung 29 were planted every 20 rows and around each plot as spreader rows to increase the uniformity of the stripe rust inoculum across the trials. The accessions were inoculated with urediniospores of uniformly mixed *Pst* isolates (CYR32, CYR33, CYR34, G22–14, Su11–4, Su11–5, and Su11–7) (Table 7) [4, 42, 63–66] when plants had developed to the shooting stage in January. The *Pst* isolates were the prevalent races in China. The stripe rust reaction response collection was initiated when SY95–71 and Taichung 29 displayed DS levels of up to 80%. The stripe rust response was evaluated three times, once per week. We scored IT using the 0–4 scale described by Stakman et al. (1962) [67], as follows: highly resistant (HR, 0–1), moderately resistant (MR, 2), moderately susceptible (MS, 3), and susceptible (S, 4). The DS was scored as percentage of infected leaf area (0, 5, 10, 20, 40, 60, 80%, or 100%) according to the rules for Monitoring and Forecast of wheat stripe rust (National Standard of the People’s Republic of China, GB/T 15795–2011).

**Table 7:** The avirulence /virulence formula of the *Pst* isolates used in this study

Race	Avirulence/Virulence formula	Reference
CYR34	*Yr5, Yr15, Yr32, YrTr1*/*Yr1, Yr2, Yr3, Yr4, Yr6, Yr7, Yr8, Yr9, Yr10, Yr11, Yr12, Yr13, Yr14, Yr16, Yr17, Yr18, Yr19, Yr25, Yr24, Yr26, Yr27, Yr28, Yr29, Yr30, Yr31, Yr32, Yr43, Yr44, YrExp2, YrA, YrSk, YrSp*	[4, 63, 64]
CYR33	*Yr5, Yr10, Yr15, Yr10, Yr19, Yr24, Yr26, Yr32, YrTr1*/*Yr1, Yr2, Yr3, Yr4, Yr6, Yr7, Yr8, Yr9, Yr11, Yr12, Yr13, Yr14, Yr16, Yr17, Yr18, Yr25, Yr27, Yr28, Yr29, Yr30, Yr31, Yr32, Yr43, Yr44, YrExp2, YrA, YrSk, YrSp*	[42, 63, 64]
CYR32	*Yr5, Yr10, Yr15, Yr10, Yr19, Yr24, Yr26, Yr32, YrTr1*/*Yr1, Yr2, Yr3, Yr4, Yr6, Yr7, Yr8, Yr9, Yr11, Yr12, Yr13, Yr14, Yr16, Yr17, Yr18, Yr25, Yr27, Yr28, Yr29, Yr30, Yr31, Yr32, Yr43, Yr44, YrExp2, YrA, YrSk, YrSp*	[42, 63, 64]
G22–14	*Yr3b, Yr4b, Yr5, Yr15, Yr17, Yr18*/*Yr1, Yr2, Yr3, Yr4, Yr6, Yr7, Yr8, Yr9, Yr10, Yr11, Yr12, Yr13, Yr14, Yr9, Yr10, Yr16, Yr19, Yr20, Yr24, Yr26*	[65, 66]
Su11–4	*Yr5, Yr10, Yr11, Yr12, Yr15, Yr17, Yr19, Yr24, Yr26, Yr27, Yr32, YrSp, YrTr1*/*Yr1, Yr2, Yr3, Yr4, Yr6, Yr7, Yr8, Yr9, Yr13, Yr14, Yr16, Yr18, Yr28, Yr29, Yr31, Yr43, Yr44, YrExp2*	[42, 64]
Su11–5	*Yr5, Yr10, Yr12, Yr13, Yr15, Yr17, Yr19, Yr24, Yr26, Yr27, Yr32, YrTr1*/*Yr1, Yr2, Yr3, Yr4, Yr6, Yr7, Yr8, Yr9, Yr11, Yr14, Yr16, Yr18, Yr28, Yr29, Yr31, Yr43, Yr44, YrExp2, YrSp*	[42, 64]
Su11–7	*Yr5, Yr10, Yr13, Yr15, Yr17, Yr19, Yr24, Yr26, Yr27, Yr32, YrTr1*/*Yr1, Yr2, Yr3, Yr4, Yr6, Yr7, Yr8, Yr9, Yr11, Yr12, Yr14, Yr16, Yr17, Yr18, Yr28, Yr29, Yr31, Yr43, Yr44, YrExp2, YrSp*	[42, 64]

*Pst*, *Puccinia striiformis* f. sp. *tritici*

### Phenotypic data analyses

To eliminate the environmental impact on stripe rust, we used a linear model with random effects for variance components to calculate the BLUP values with the lme4 package in R [68]. Based on the BLUP values, the analysis of variance and a correlation analysis with the Pearson’s method were computed using SPSS 20.0 (IBM Corp., Armonk, NY, USA). The broad-sense heritability (*H*^*2*^) estimates for IT and DS were calculated across six test environments using the lme4 package [68] with the formula *H*^*2*^ = V_G_/(V_G_ + V_E_), where V_G_ and V_E_ represent the genotypic and environmental variances, respectively [69]. The phenotypic variations were confirmed by the value range, average values, standard deviation (STDEV), and coefficient of variation (CV) of all traits in six environments and BLUP values. The *H′* was calculated for IT and DS using BLUP values [70].

### Genotyping and molecular diversity analyses

For each accession, genomic DNA was extracted from mixed leaves of five 1-week-old seedlings using a plant DNA kit (Biofit Co., Chengdu, China). The 244 accessions were genotyped using the 55 K SNP microarray (Affymetrix Axiom Wheat55K) at the China Golden Marker Biotechnology Company Ltd. (Beijing, China). Markers with missing values of ≤10% and MAFs of ≥5% were selected for the linkage analysis. Statistical analyses of PIC, major allele frequency, and gene diversity were performed using the software POWERMARKER v3.25 [71] to determine genetic diversity. These indices of genetic diversity were used to compare the extent of molecular diversity among different sub-genomes and chromosomes. The same comparisons were performed between landraces and cultivars, as well as among different classifications.

### Population structure, kinship and LD analyses

To analyze the population structure (Q-matrix), Bayesian model-based clustering was performed in STRUCTURE v2.3.4 using 44,059 SNP markers (missing ≤10% and MAF ≥ 5%) with the ΔK method [72, 73]. In total, five independent STRUCTURE runs were performed with K values from 2 to 10 using the admixture model with 100,000 replicates for burn-in length and 100,000 replicates for Markov chain Monte Carlo iterations. The optimal K value was chosen based on the ΔK method [73], which was implemented using the web-based informatics tool STRUCTURE HARVESTER [74].

The identity-by-state relative K-matrix was estimated between pairs of accessions as a measure of relatedness. Heat maps were generated with the pheatmap R package v1.0.8 [75] based on the K-matrix. The pairwise measure of LD was estimated as squared allele frequency correlation (*r*^*2*^) values between pairs of intra-chromosomal markers with known chromosomal positions. Significant pair-wise markers were chosen using the criteria pDiseq < 0.001 and *r*^*2*^ > 0.1. The LD decay plot and half-decay distance were generated from the *r*^*2*^ value and the distance between markers using ggplot2 package with R [76]. All the high-confidence associated loci that were included in the half-decay distance regions of the same chromosome were defined as the same QTL block. The K-matrix and LD were analyzed using TASSEL v5.2.38 [77].

### Genome-wide association analyses

A GWAS was performed on 244 wheat accessions with the software TASSEL v5.2.38 based on a mixed linear model with Q and K as covariates [77–79]. The marker-trait associations for responses to stripe rust were identified from 44,059 SNP markers using IT and DS values collected from six test environments. The significant association loci were considered if the *p*-value < 0.001. The associated loci were visualized with a Manhattan plot using ggplot2 package with R [76]. To obtain confident marker-trait associations, highly associated loci detected in at least three environments were selected for further analyses. The statistical analysis for favorable alleles was performed using SPSS 20.0 (IBM Corp).

### Analyses of high-confidence significant associated resistance loci

To determine whether our associated loci were novel, we compared the locations of QTLs in this study with those of previously reported *Yr* genes and QTLs based on an integrated map. This map, included the 78 permanently named *Yr* genes, 67 temporarily designated *Yr* genes and 327 QTLs, and was constructed by Wang and Chen (2017) [25] using the software BioMercator v4.2 [80]. Some correlations between QTLs in this study and reported *Yr* genes or QTLs could not be determined because the associated markers were absent from the consensus map. The physical position comparison was carried out using the Chinese Spring reference (IWGSC RefSeq v1.0) [26] with BLAST+ v2.7.1 [81].

### Analyses of presumptive candidate genes

Here, we identified the gene sequences of potential novel QTLs based on the Chinese Spring reference genome (IWGSC RefSeq v1.0) and RefSeq Annotation v1.1 [26]. Collinear alignment with *A. thaliana*, *Brachypodium distachyon*, *O. sativa* L. ssp. *japonica*, *Z. mays* L. and *Hordeum vulgare* was carried out to determine candidate genes. The candidate genes were obtained using online BLAST at the EnsemblPlants website (https://plants.ensembl.org/Multi/Tools/Blast?db=core) with default parameters and using DIAMOND blastx [82] (E value < 10^− 10^ and bitscore > 60) based on the SWISS-PROT database [83].

## Additional files

Additional file 1: The 244 wheat accessions used in this study and the evaluation of their infection type (IT) and disease Severity (DS) in six environments. (XLSX 36 kb)

Additional file 2: The distribution of infection type (IT) and disease severity (DS) for stripe rust between landraces and cultivars in adult-plant stage based on BLUP values. (XLSX 10 kb)

Additional file 3: Summary of genetic diversity between landraces and cultivars with 44,059 SNP markers. (XLSX 10 kb)

Additional file 4: The phenotypic and genetic diversity analyses of 244 wheat accessions in two sub-populations. (XLSX 9 kb)

Additional file 5: The distribution of the favorable alleles in 244 wheat accessions. (XLSX 32 kb)

Additional file 6: The Pearson correlation analyses between favorable allele number and the stripe rust response. (XLSX 9 kb)

Additional file 7: The putative candidate genes of two potentially novel QTLs. (XLSX 14 kb)

## Abbreviations

ABC: ATP-binding cassette
APR: Adult plant resistance
ASR: All-stage resistance
BLUP: Best linear unbiased prediction
CV: Coefficient of variation
DS: Disease severity
GWAS: Genome-wide association study
HR: Highly resistance
*H‘*: Shannon-Weaver diversity index.
*H*^*2*^: Broad sense heritability
IT: Infection type
LD: Linkage disequilibrium
MAF: Minor allele frequency
Mb: Million base pairs
MR: Moderately resistance
MS: Moderately susceptible
PIC: Polymorphism information content index
*Pst*: *Puccinia striiformis* f. sp. *tritici*
PVE: Phenotypic variation explained
QTL: Quantitative trait loci
S: Susceptible
SNP: Single nucleotide polymorphism
SP: Sub-population
STDEV: Standard deviation
*Yr*: Yellow rust resistance
